# Enhancing outcomes in Langendorff-perfused rodent hearts through perfusion parameter optimization

**DOI:** 10.1038/s41598-025-00159-3

**Published:** 2025-05-07

**Authors:** Maya Bolger-Chen, Manuela Lopera Higuita, Casie A. Pendexter, Mohammadreza Mojoudi, Korkut Uygun, Shannon N. Tessier

**Affiliations:** Center for Engineering in Medicine and Surgery, Massachusetts General Hospital, Harvard Medical School, and Shriners Children’s Boston, Boston, MA USA

**Keywords:** Cardiology, Diseases

## Abstract

Despite important advancements in addressing cardiovascular diseases (CVDs), there has been an overall lack of progress in the field, leading to a slower decline in the rate of CVDs related deaths, and even an increase for some risk groups (e.g. increase in stroke mortality) exacerbated by an aging and obese population. While a multi-faceted problem, this deceleration may be influenced by the preferred model systems utilized in translation research. Cardiac cell lines, although easier to handle, lack biological accuracy due to the unnatural modifications required for successful culture and may not recapitulate complex 3-dimensional structural and environmental factors. At the same time, whole animal experimentation provides unwanted complexity during initial scientific development. Alternatively, ex vivo perfusion of isolated rodent hearts provides the needed biological accuracy with decreased organismal complexity. This platform facilitates the evaluation of the isolated heart, without neuro-reflexes and/or humoral contributions, unveiling the direct effects of stimuli in heart function/homeostasis. This manuscript leverages the wide array of perfusion parameters (i.e. perfusate, flow rate, coronary pressures), to demonstrate the capability of ex vivo heart perfusion protocols to accommodate a large range of experimental needs. Through this work, it was determined that the use of physiological perfusion pressures leads to increased left ventricular (LV) pressures but results in a loss of function over time, making it ideal conditions for organ assessment. Conversely, lower-than-physiological perfusion pressures lead to decreased LV pressures but prevent loss of function over time, which is preferable when longer perfusion times are relevant to experimental needs. Similarly, the use of adenosine as a pharmacological intervention was found to decrease both edema formation and inflammatory responses. In contrast, the use of packed red blood cells as oxygen carriers appears to induce a pro-inflammatory response and cause greater cardiac damage, particularly when combined with low perfusion pressures.

## Introduction

Despite important advancements in cardiovascular diseases (CVDs) research, CVDs remain the leading cause of death globally, accounting for > 900,000 deaths per year in the United States alone^1,2^. These disquieting figures highlight the absolute need for increased advancements in CVDs research^3^. Unfortunately, the field has seen a lack of progress, leading to a slower decline in the rate of CVDs related deaths and even an increase for some risk groups (e.g. increase in stroke mortality)^4^. This deceleration of progress can be partly attributed to the lack of accurate and predictive model systems for early-stage scientific testing in the cardiovascular field.

Early basic research is heavily dependent on in vitro cell culture methods as initial testing grounds due to its simplicity, low cost, controllability and reproducibility^5^. However, the field of CVDs is severely underserved by cellular model systems^6^. In vivo models of CVDs offer high physiological relevance but introduce unnecessary complexity in early research stages^7,8^. Alternatively, rodent ex vivo heart perfusion (Langendorff perfusion) offers the much-needed middle ground complexity, providing several advantages over traditional in vitro and in vivo models. It preserves the intricate interplay of cardiac cells and their immediate microenvironment while eliminating the cofounding effects of neuro-reflexes, humoral and systemic contributions^9^. Furthermore, ex vivo heart perfusion is a highly dynamic and modifiable technique allowing the precise manipulation and monitoring of experimental settings. This flexibility facilitates the access to a wide array of cardiac processes, including electrophysiology, contractility, metabolism, and responses to pharmacological interventions^10–12^.

However, despite centuries since its inception and decades of implementation, the field has completely exploited the most versatile aspect of ex vivo heart perfusion: the wide array of easily adjustable perfusion parameters (i.e. coronary flow, coronary perfusion pressures, circulating solution, temperature, etc.)^13,14^. The ability to modify these parameters independently makes this technique immensely customizable, allowing an impressive degree of tailoring toward specific scientific needs. Despite this capability, the field has historically utilized perfusion parameters that strictly mimic physiological conditions (i.e. perfusion pressures between 60 and 80 mmHg, perfusate temperature at 37 °C, etc.) without considering specific experimental needs^15–18^. For instance, previous work by our group demonstrated low-pressure (30–35 mmHg) perfusion are more suitable for preservation purposes or for experiments that require longer perfusion times^14^. In contrast, high-pressure perfusions are more suitable for functional assessment purposes.

In effect, this manuscript aims to further explore and determine the effects that easily adjustable perfusion parameters (i.e. high vs. low perfusion pressures, cellular vs. acellular perfusate, ionotropes vs. no ionotropes, vasodilators vs. no vasodilators) have on cardiac viability, functionality, and injury markers. It also seeks to establish a baseline of knowledge from which researchers can determine the perfusion parameters that maximize the technique’s potential based on experimental needs. In doing so, we highlight the capability of ex vivo machine perfusion to accommodate a large range of experimental scenarios through thoughtful selection of perfusion parameters.

## Methods

### Ethical statement

All research complies with the ethical standards and regulations set forth by National Research Council guidelines and in the experimental protocol 2018N000094 approved by the Institutional Animal Care and Use Committee (IACUC) of Massachusetts General Hospital (Boston, MA, USA) and the ARRIVE guidelines.

### Animals

Both male and female Lewis rats (250–300 g body weight, 8–9 weeks old) were purchased from Charles River Laboratories (Boston, MA, USA). All animals were maintained in accordance with National Research Council guidelines and were approved by the Institutional Animal Care and Use Committee (IACUC) at Massachusetts General Hospital (Boston, MA, USA). A total of 8 animals were ran for each group, 4 to obtain biochemical, inflammatory and injury markers and 4 for functional assay involving an intraventricular balloon.

### Heart procurement

The animals were anesthetized with 3% isoflurane (Piramal Critical Care Inc., Bethlehem, PA, USA). Male rats were heparinized through an injection (30 U) through the dorsal penile vein. Female rats were heparinized after abdominal incision by injection in the vena cava. The surgical site was shaved, and a horizontal midline incision was made in the abdominal wall. The sternum was retracted cranially using Halstead forceps and the abdominal organs were displaced to expose the portal vein. The portal vein was cannulated using a 16 g angiocath and the inferior vena cava/abdominal aorta were cut to vent. Heparinized saline (60mL, 33.3 U/mL) was flushed through the portal vein, maintaining the flush pressures around 10 mmHg. The animal was euthanized via exsanguination. Upon completion of the portal flush, the diaphragm was cut on both sides of the sternum exposing the thoracic cavity. The heart was removed from the thoracic cavity and immediately placed in 4ºC heparinized saline to arrest the organ. The aorta was exposed, cleaned of any remaining connective tissue, and the brachiocephalic artery branch on the aorta was localized. The aorta was cut halfway below the branch and cannulated with a 14 g angiocath (BD, Franklin Lakes, NJ, USA) primed with saline.

### Heart perfusion

All chemicals were purchased from Sigma Aldrich unless stated otherwise. All hearts were retrogradely perfused for 4 h in a double jacketed organ chamber (Radnoti LLC, Covina, CA, USA) at 37 °C with 75mL of base perfusate containing 0.96% Krebs-Henseleit Buffer, 0.125 mM Dextran, 25 mM Sodium Bicarbonate, 1.054 mM Bovine Serum Albumin, 1% Pen Strep (10,00 U/mL), 0.13% Insulin (100 U/mL), 0.02% Hydrocortisone (50 mg/mL), 0.5% Heparin (1000 U/mL), and 2.75 mM Calcium Chloride. Perfusate was oxygenated with 95% O2–5% CO2 using a porous silicone coil oxygenator (Radnoti LLC, Covina, CA, USA) and circulated through a bubble trap (Radnoti LLC, Covina, CA, USA) to avoid insertion of air into the coronaries. After procurement, hearts were weighted and connected to the perfusion system starting at a flow rate of 1mL/min. Flow rates were increased by 0.2mL/min quickly, while monitoring the coronary pressures until the desired pressure was reached. A total of six experimental groups (Table 1, *n* = 4 each) were tested to determine the effects of different perfusion parameters on viability, functionality, and injury markers.

**Table 1:** Experimental groups.

Group	Pressure	pRBCs	Drip
Cellular high-pressure	High	Yes	No
Cellular low-pressure	Low	Yes	No
Acellular high-pressure Adeno	High	No	Adenosine
Acellular low pressure Adeno	Low	No	Adenosine
Acellular low-pressure Adeno/Epi	Low	No	Adenosine + Epinephrine
Acellular low-pressure no treatment	Low	No	No

Hearts were retrogradely perfused at high (60–80 mmHg) or low pressures (30–35 mmHg), with or without packed red blood cells, and with or without a direct drug drip. Packed red blood cells (pRBCs) were isolated by centrifuging (3000 rpm) 10–12 mL of whole rat blood (obtained via cardiac puncture of donor animals) for 10 min. After initial centrifugation, the plasma/buffy coat layer was removed, followed by resuspension of the pRBCs in base perfusate without CaCl at a 1:1 ratio (e.g. 5 mL pRBCs: 5mL perfusate). pRBCs were washed a total of three times. After the last wash, the pRBCs were added to the perfusion system and allowed to distribute homogeneously before connecting the heart. Hematocrit levels were measured using a hematology analyzer (Sysmex, Kobe, Hyogo, Japan) and ranged from 5 to 7%. Hearts in three groups were treated with a direct drug drip (Table 1), delivered via a syringe pump connected to the system as close as possible to the organ. Adenosine was diluted in 50 mL of perfusate and delivered at a rate of  1.66 ug/min. Epinephrine was diluted in 50mL of saline and delivered at a rate of 0.1 mcg/kg/min.

### Viability assessment

Oxygen and lactate measurements were quantified from perfusate samples via a blood gas analyzer (Siemens Medical Solutions, Malvern, PA, USA) 20 min after initiating perfusion and every hour thereafter. The oxygen uptake rate (OUR) was determined following the below formulas:

$$\begin{aligned} {\text{Blood perfusions}} & -\left( {a{O_2}*Flow*\left( {aortic~p{O_2} - venous~p{O_2}} \right)} \right.~~+Hb \\ & \;\;*cHb*\left. {Flow*\left( {\frac{{aortic~s{O_2} - venous~s{O_2}}}{{100}}} \right)} \right)*~\frac{1}{{Heart~Weight}} \\ \end{aligned}$$

$${\text{Acellular perfusions}}-\left( {a{O_2}*AorticFlow*\left( {aortic~p{O_2} - venous~p{O_2}} \right)} \right)~*~\frac{1}{{Heart~Weight}}$$

Where aO2 is oxygen solubility coefficient (0.00314 µLO_2_/mmHgO_2_/mL); Aortic pO2 is the partial oxygen pressure (mmHg) of perfusate going to the coronaries; Venous pO2 is partial oxygen pressure (mmHg) of perfusate coming out of the coronary sinus; Flow is flow rate (ml/min); aortic sO_2_ is hemoglobin saturation (%) of perfusate going into the coronaries; venous sO2 is hemoglobin saturation (%) of perfusate coming out of the coronary sinus; cHb is hemoglobin oxygen-binding capacity (1.34 ml O_2_/g); Hb is hemoglobin (g/ mL)^19^.

Hearts were weighed immediately before being placed on the perfusion system on a precision weight scale and immediately after removal from the system. These values were utilized to calculate percent weight gain, a proxy for edema. The energetic status of cardiac cells was calculated via the energy charge of the adenylate system^20^. The metabolic cofactors (ATP, AMP, and ADP) were obtained via Mass Spectrometry, as described previously^21^. Briefly, the apex of the hearts was cut transversely at the end of the perfusion time, flash-frozen in liquid nitrogen, and stored at – 80 °C. Flash-frozen tissue was homogenized and analyzed using a 3200 triple quadrupole liquid chromatography-mass spectrometry system (AB Sciex, Toronto, Canada). The remaining portion of the heart was fixed in 5% formaldehyde for a minimum of 24 h, transferred to 70% Ethanol, and embedded in paraffin. Fixed samples were subsequently stained with hematoxylin and eosin.

### Injury and pro-inflammatory assessment

Troponin I was measured from outflow perfusate samples using cTnI i-STAT cartridges and blood-gas analyzer (Abbott, Chicago, IL, USA). Troponin T, creatine kinase muscle (CKM) and myosin light chain 3 (MYL3, MilliporeSigma, Burlington, MA), as well as intracellular adhesion molecule (ICAM), interleukin 6 (IL-6) and chemokine (C-X-C motif) ligand 1 (CXLC1, R&D systems, Minneapolis, MN) were measured via bead-based immunoassay (Luminex), following the manufacturer’s instructions. Outflow perfusate samples at 20 min (T0) and 4 h (T5) were concentrated via 30 kDa centrifugal filters by centrifuging for 1 h, 4000 rpm at 4 °C. The concentrate was store at 4 °C until ready to use. The wash through was further concentrated via a 3 kDa centrifugal filter by centrifugation with same settings. The concentrates from both filters were combined and utilized in the immunoassay. Analyte readings were normalized by subtracting T0 from T5 readings.

### Functional assessment

Vascular resistance was calculated by dividing the coronary pressure by flow rate and corrected for weight of the heart after procurement. Left ventricular pressures were acquired via a pressure balloon (Radnoti LLC, Covina, CA, USA) attached to a fluid-filled pressure sensor (iWorx, Dover, NH). Briefly, after 20 min of perfusion time, a small orifice was made in the left atrium. The balloon was inserted through this orifice, into the atrium and advanced through the mitral valve to reach the left ventricle. Once situated, the balloon was filled with saline until pressure during diastole read 0 mmHg. The left ventricular pressure was continuously recorded using Labscribe software (iWorx, Dover, NH, USA), and the data processed with MATLAB (The MathWorks Inc, Natick, MA). Heart rate was calculated by counting the number of peaks per second from the balloon recordings and multiplied by 60 to obtain beats per minute. The resulting heart rate numbers were averaged over a period of 10 s and graphed overtime.

### Statistical analysis

All data were analyzed for outliers using the ROUT method with Q = 1%, statistical outliers were excluded from data graphs. Over-time data were analyzed using repeated measures two-way ANOVA and Tukey-Kramer HSD post-hoc analysis on standard least squares means. Column data were analyzed via Wilcoxon/Kruskal-Wallis Test with Dunn post-hoc analysis on non-parametric medians. All data are expressed as median ± interquartile range (IQR). Statistical significance is defined at *p* < 0.05.

## Results

The results in this study have been arranged to facilitate the determination of the effects of high vs. low perfusion pressures, as well as the differences between cellular and acellular perfusates. As important effects on graft homeostasis were observed with the use of high perfusion pressures and oxygen carriers, acellular low-pressure perfusion was employed to test the effects of pharmacological interventions (i.e. adenosine, epinephrine) without the confounding influence of damage caused by the perfusion parameters.

### Perfusion without oxygen carriers results in lower oxygen consumption rate but does not incur in a significant decrease of adenylated energy charge

**Fig. 1:**
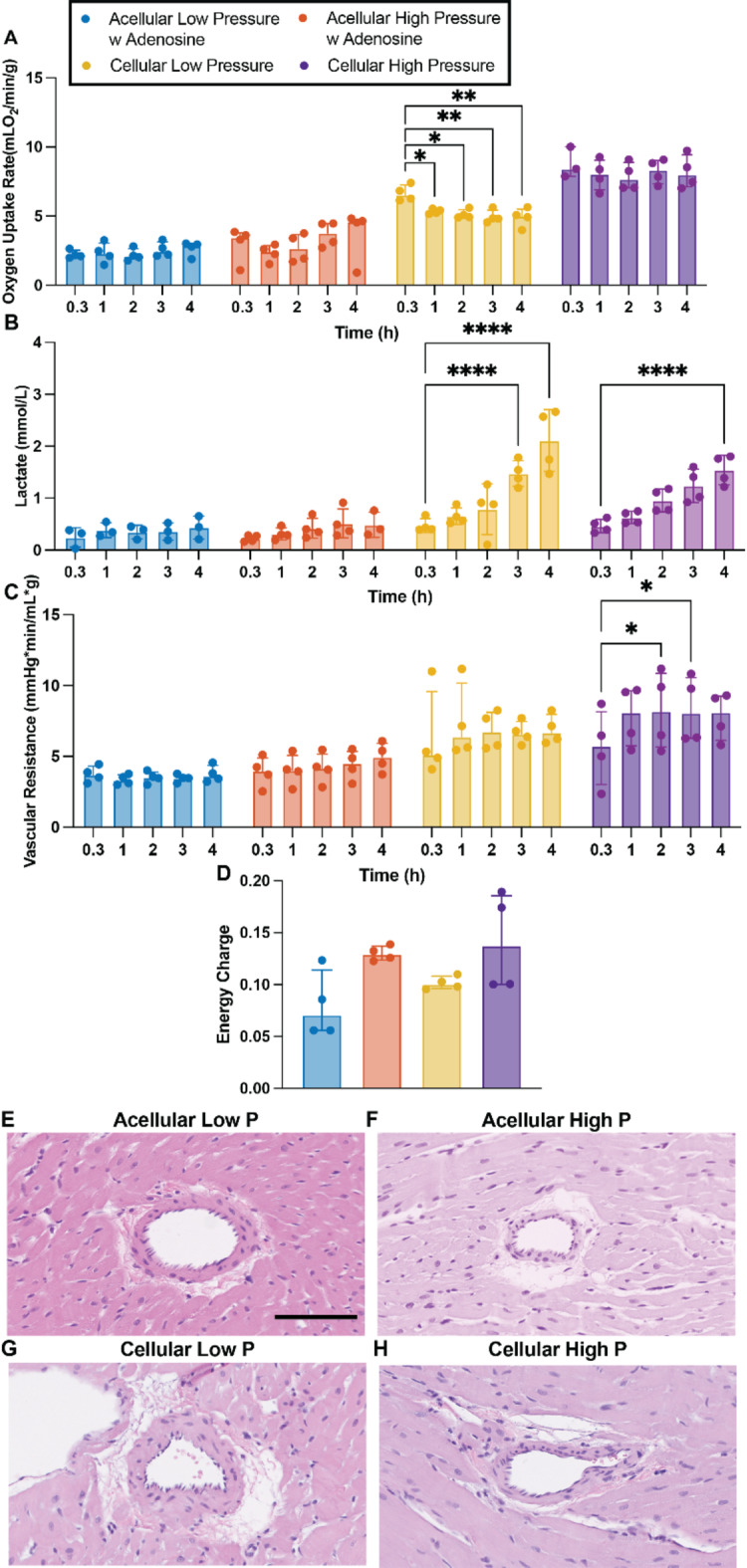
Viability markers of hearts perfused with or without pRBCs at low (30–35 mmHg) and higher (60–80 mmHg) pressures. Oxygen uptake rate (A), lactate accumulation over perfusion time (B), vascular resistance (C), adenylated energy charge (D), representative H&E images of coronary arteries (E-H). Scale bar 50 μm. Data presented as median ± interquartile range. Wilcoxon/Kruskal-Wallis Test with Dunn post-hoc analysis on non-parametric medians. Repeated measures two-way ANOVA and Tukey-Kramer HSD post-hoc analysis on standard least squares means. * = *p* < 0.05, ** = *p* ≤ 0.01, **** = *p* ≤ 0.0001.

The use of oxygen carriers in normothermic machine perfusion (NMP) has been considered a necessity due to the prevailing belief that perfusates without these provide inefficient oxygen delivery and maintains the organ in a state of moderate ischemia^22,23^. To address this, this study employed perfusates, both with (cellular perfusate) and without (acellular perfusate) oxygen carriers (packed red blood cells - pRBCs), to investigate their respective impact on oxygen delivery and metabolic support during retrograde perfusion. This resulted in hearts perfused with acellular perfusate displaying lower rate of oxygen consumption (Fig. 1A), than hearts perfused with cellular perfusates, with this difference not translating into significant changes in the levels of adenylated energy charge (Fig. 1D). The use of pRBCs, irrespective of perfusion pressure, did result in an overtime, significant increase in lactate levels, a phenomenon not seen in hearts perfused with acellular perfusate (Fig. 1B). Similarly, coronary vascular resistance, a metric of vascular health, was higher in hearts perfused with cellular perfusate when compared with hearts perfused with acellular perfusate (Fig. 1C). Interestingly, however, the endothelial cell lining in the coronary arteries was retained irrespective of perfusion parameters (Fig. 1E–H).

### Coronary perfusion pressures, more so than oxygen carrying capacity, drive left ventricular pressures

**Fig. 2:**
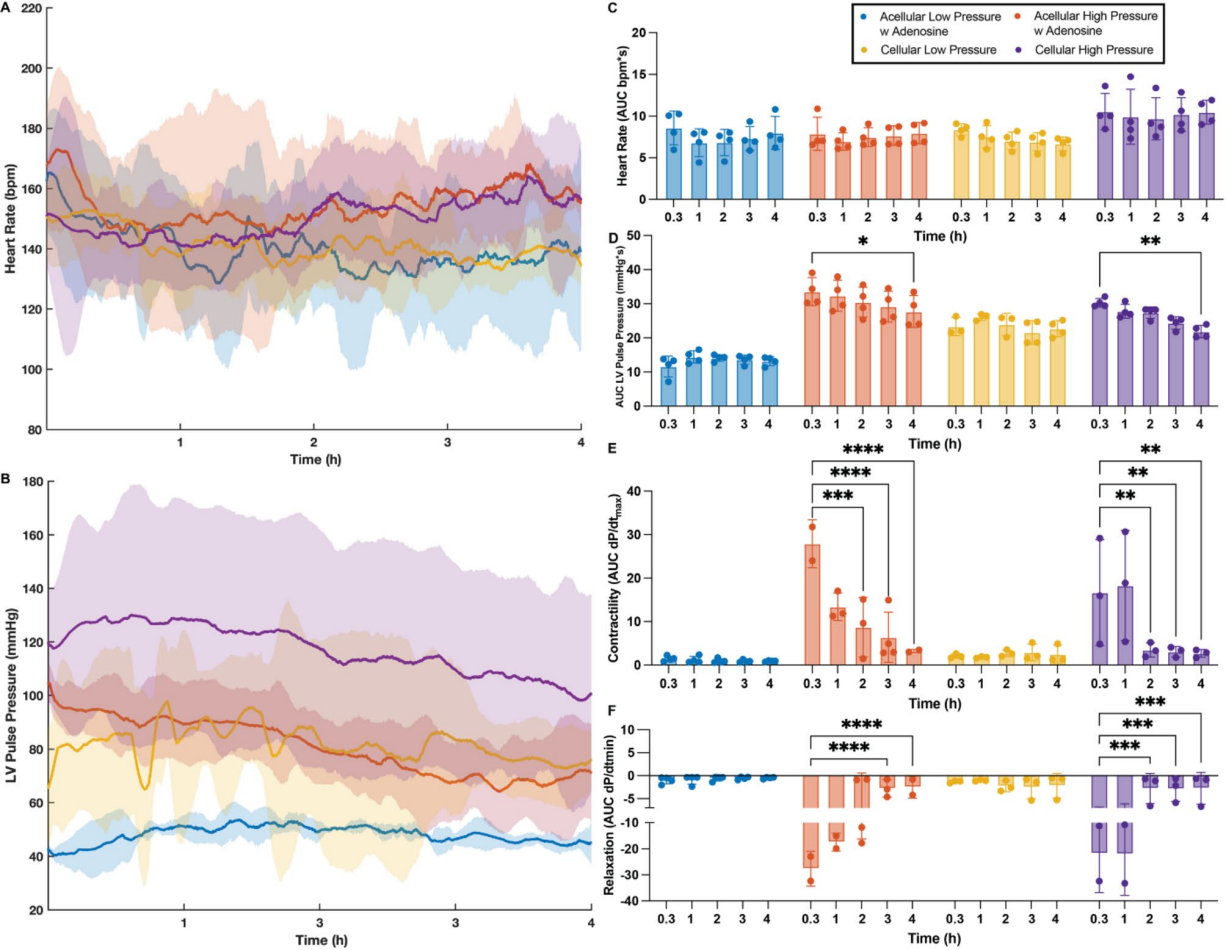
Influence of cellularity and perfusion pressures in cardiac functional markers. Heart rate, obtained from intraventricular balloon recordings. Solid line indicates median of the experimental replicates, the shaded area indicates interquartile range (A), maximum systolic pressure over time, denoted as left ventricular pulse pressure (LVPP). Solid line indicates median of the experimental replicates, the shaded area indicates interquartile range (B), area under the curve (AUC) of heart rate data for every hour of perfusion (C), the area under the LVPP curve (AUC) for every hour of perfusion (D), cardiac muscle contractility quantified from the maximum derivative of the pressure pulse (E), cardiac muscle relaxation quantified from the minimum derivative of the pressure pulse (F). Repeated measures two-way ANOVA and Tukey-Kramer HSD post-hoc analysis on standard least squares means. All data are expressed as median ± interquartile range. * = *p* < 0.05, ** = *p* ≤ 0.01, *** = *p* ≤ 0.001, **** = *p* ≤ 0.0001.

Perfusion pressures directly correlate with the pressures experienced by the coronary vasculature, which are known to directly control cardiac performance^24–27^. Physiological pressures (60–80 mmHg) resulted in slightly higher heart rate (Fig. 2A and C) and left ventricular pulse pressure (Fig. 2B and D) when compared to low pressure groups. Interestingly, no difference in myocardial contractility (Fig. 2E, *p* = 0.242) and relaxation (Fig. 2F, *p* = 0.686) were present between hearts perfused at high pressures, irrespective of perfusate cellularity, whereas hearts perfused at low pressures demonstrated significantly lower contractility and relaxation. The increased LV pressures produced by hearts perfused at high pressures, irrespective of perfusate cellularity, led to an overtime loss of function, resulting in a significant decrease in LV pulse pressure (Fig. 2D), contractility (Fig. 2E) and relaxation (Fig. 2F) at different times into the perfusion when compared to their initial values at 20 min. Interestingly, despite the difference in oxygen carrying capacity between high-pressure hearts perfused with and without pRBCs (Fig. 1A, *p* < 0.0001) the overall ventricular pressures by hearts in both groups and the loss of function trend is remarkably similar. Hearts in both groups produced similar magnitudes of LV pulse pressure (p_20min_ = 0.393), contractility (p_20min_ = 0.043) and relaxation (p_20min_ = 0.582) during the initial stages of perfusion and thereafter, and even experiencing similar loss of function overtime maintaining all functional metrics statistically the same (**LV pulse pressure –** p_4h_ = 0.999, **Contractility –** p_4h_ > 0.999, **Relaxation –** p_4h_ = 0.17).

### Langendorff perfusion at higher pressures (60–80 mmHg) lead to increased edema and moderate organ damage

**Fig. 3:**
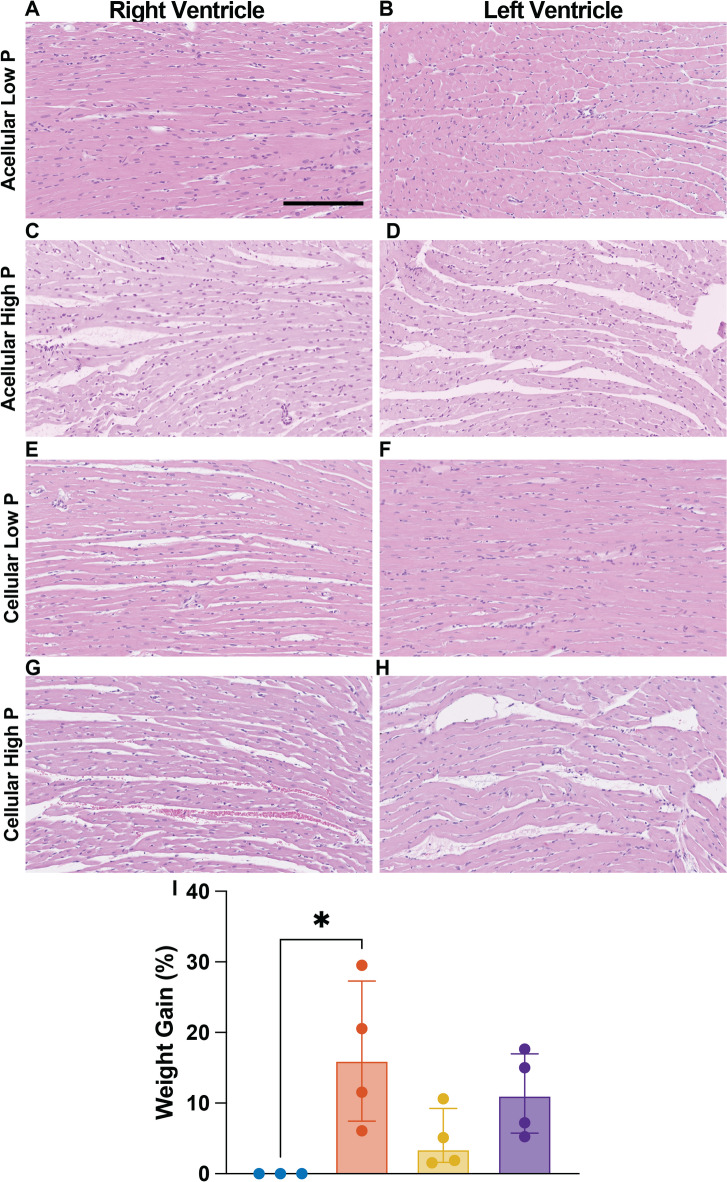
Effects of high and low prefusion pressure in cardiac edema. Representative H&E images of right and left ventricle (A-H) and percent weight gain (I). Scale bar ventricle images 200 μm. Wilcoxon/Kruskal-Wallis Test with Dunn post-hoc analysis on non-parametric medians. All data are expressed as median ± interquartile range. * = *p* < 0.05.

**Fig. 4:**
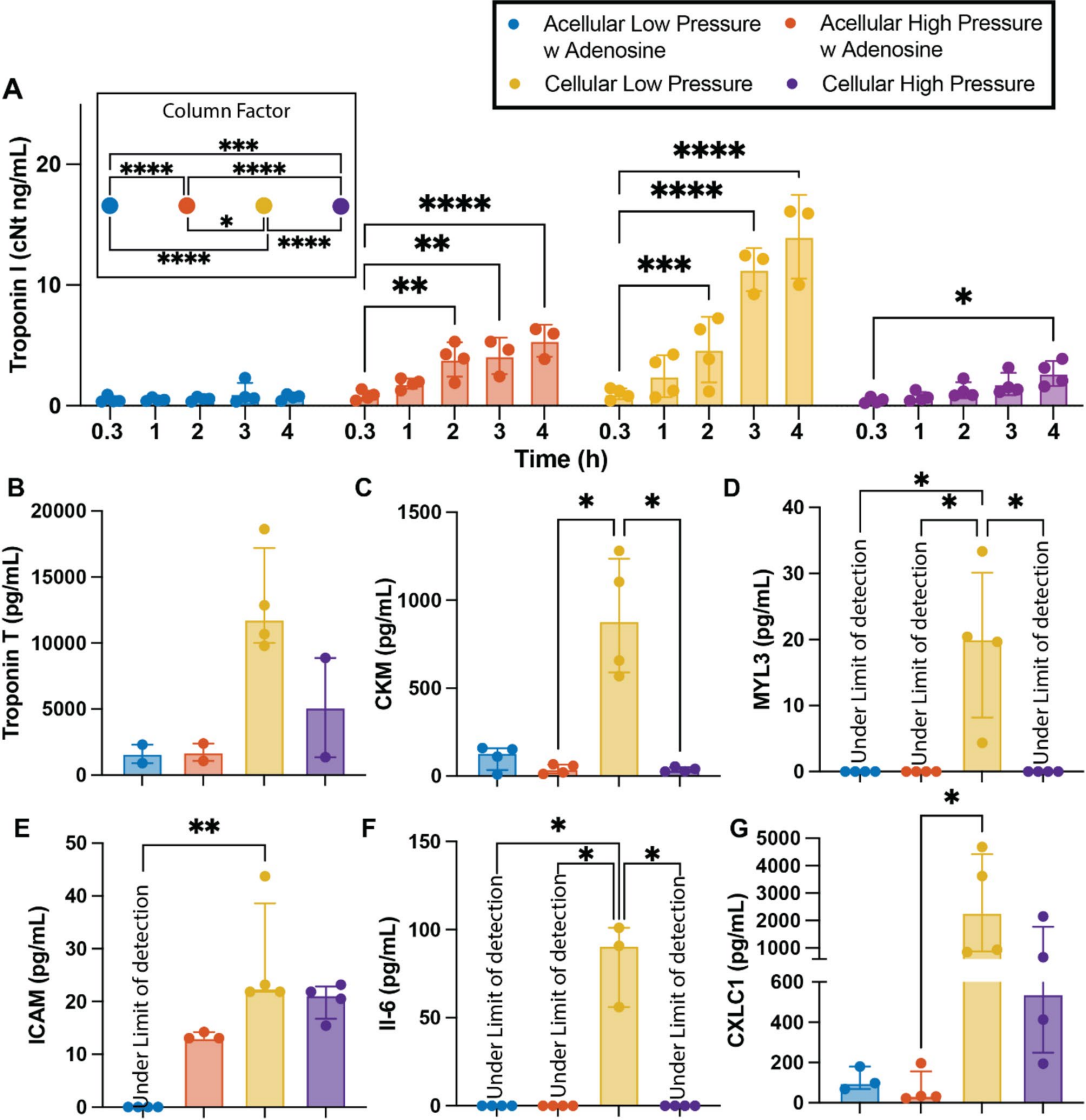
Influence of cellularity and perfusion pressures in cardiac injury and inflammatory markers. Concentration of Troponin I in the perfusate over time (A). Concentration of cardiac myocyte-associated proteins, a proxy for cardiac injury, quantified from the perfusate after 4 h (240 min) of perfusion, including Troponin T (B), creatine kinase – CKM (C), and myosin light chain 3 - MYL3 (D). Concentration of pro-inflammatory proteins including ICAM (E), IL-6 (F), and CXCL1 (G). Repeated measures two-way ANOVA and Tukey-Kramer HSD post-hoc analysis on standard least squares means. All data are expressed as median ± interquartile range. * = *p* < 0.05, ** = *p* ≤ 0.01, *** = *p* ≤ 0.001, **** = *p* ≤ 0.0001.

Higher perfusion pressures, while physiological, when combined with the non-physiological components of ex vivo machine perfusion, lead to slight adverse effects on organ homeostasis. Increased myocyte bundle separation was observed in, both the left and right ventricle of the hearts perfused at high-pressures (Fig. 3C, D, G, H), irrespective of perfusate cellularity. Whereas no bundle separation was observed in either ventricle of hearts perfused with acellular perfusate at low pressures (Fig. 3A, B). Interestingly, bundle separation was present in the right ventricle of hearts perfused at low pressures with pRBCs (Fig. 3E) but not in the left ventricle (Fig. 3F). The bundle separation seen in high-pressure hearts translated to higher percent weight gain (*p* = 0.002) compared to low-pressure hearts (Fig. 3I). Similarly, higher perfusion pressures led to a significant increase in Troponin I over time (**w/o pRBCs**: p_2h_ = 0.001, p_3h_ = 0.001, p_4h_ < 0.0001, **w pRBCs**: p_4h_ = 0.015), regardless of perfusate cellularity when compared to initial Troponin I values (20 min). This phenomenon was not observed in low-pressure hearts perfused without pRBCs but was, again, present in low-pressure hearts perfused with pRBCs (Fig. 4A). Despite some evidence of damage, cardiomyocytes in both ventricles of hearts from all groups seem to remain viable after 4 h of perfusion without signs of distress and/or cell death (Fig. 3A–H).

### Langendorff perfusion at low pressures (30–35 mmHg) with pRBCs result in significant organ damage and heighten inflammatory response

The combination of pRBCs with low perfusion pressures, or more likely the accompanying low flows, led to significant organ damage and inflammatory activation. Troponin I levels were statistically higher as early as 2 h (*p* = 0.001) into perfusion compared to initial values (20 min) and continued to increase over time (p_3h_ < 0.0001, p_4h_ < 0.0001, Fig. 4A). These perfusion conditions also led to an increased release of other cardiac myocyte-associated proteins into the perfusate, a proxy for cardiac injury, including troponin T (Fig. 4B), creatine kinase (CKM, Fig. 4C), and myosin light chain 3 (MYL3, Fig. 4D), as well as an increased secretion of key pro-inflammatory proteins such as ICAM (Fig. 4E), IL-6 (Fig. 4F) and CXCL1 (Fig. 4G)^28–30^. Hearts perfused with pRBCs at high pressures also experienced an increased secretion of ICAM (*p* > 0.999) and CXCL1 (*p* > 0.999) being non-statistically different than low-pressure, pRBCs hearts, while IL-6 remained under the limit of detection. Hearts perfused with acellular perfusate at low pressures experienced the lowest inflammatory activation with ICAM and IL-6 being under the limit of detection, and the secretion of CXCL1 being much lower than that of hearts perfused with pRBCs, irrespective of perfusion pressure. Perfusion with acellular perfusate at high pressures, resulted in ICAM secretion being slightly higher than that of low-pressure, acellular hearts, but slightly lower than that of hearts perfused with pRBCs. Meanwhile IL-6 remained under the limit of detection and CXCL1 secretion was statistically similar to that in acellular, low-pressure hearts.

### Continuous use of adenosine during Langendorff perfusion reduces vascular resistance, cardiac edema and pro-inflammatory activation while increasing oxygen uptake rate

**Fig. 5:**
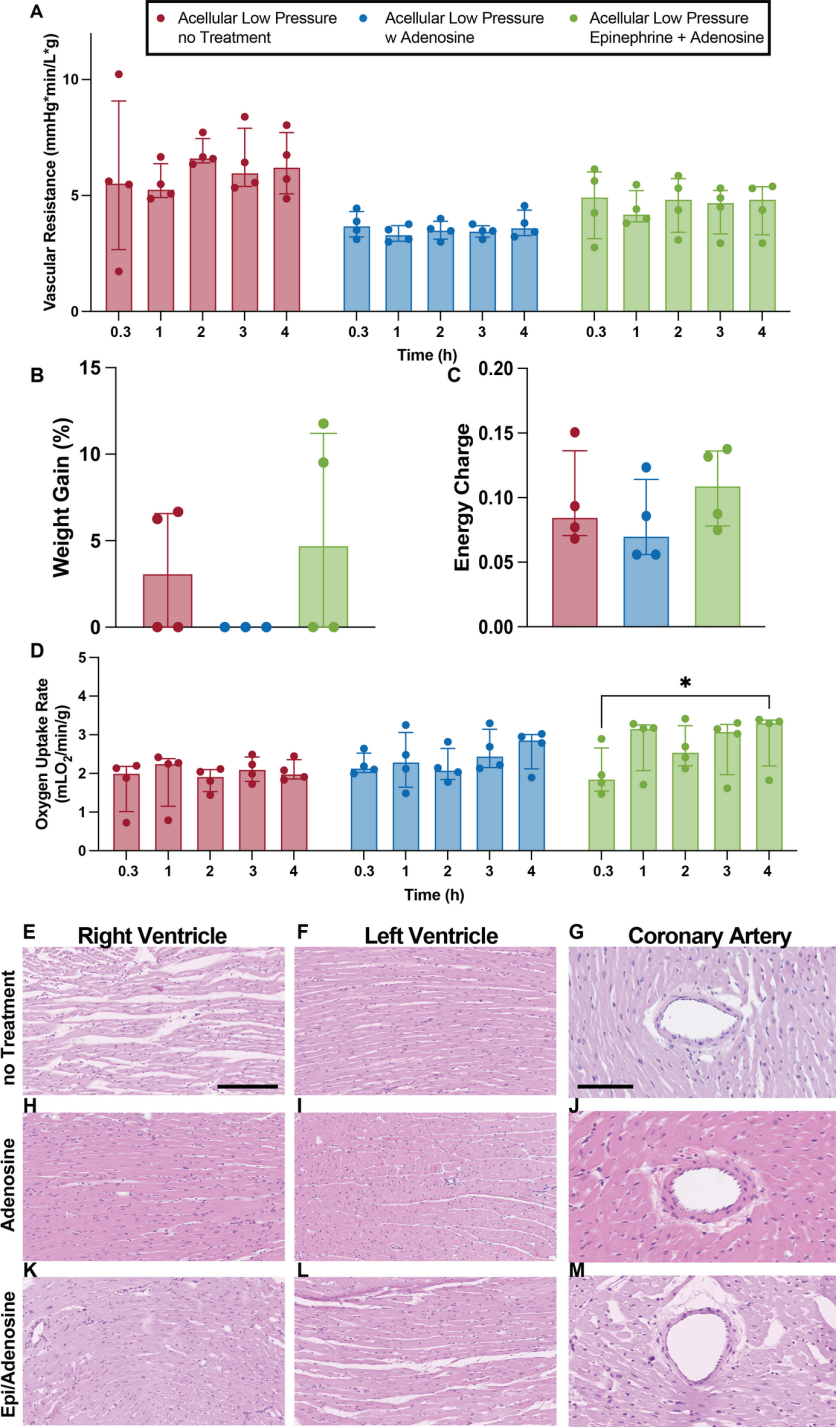
Viability markers of hearts perfused without pharmacological treatment, with infusion of adenosine only or infusion with adenosine and epinephrine. Vascular resistance (A), percent weight gain (B), adenylated energy charge (C), oxygen uptake rate D), representative H&E images of right and left ventricle and coronary arteries (E-M). Scale bar ventricle images 200 μm. Scale bar coronary arteries 50 μm. Data presented as median ± interquartile range. Wilcoxon/Kruskal-Wallis Test with Dunn post-hoc analysis on non-parametric medians. Repeated measures two-way ANOVA and Tukey-Kramer HSD post-hoc analysis on standard least squares means. * = *p* < 0.05, ** = *p* ≤ 0.01, **** = *p* ≤ 0.0001.

Among the many advantages of ex vivo machine perfusion is the ability to incorporate pharmacological treatments to better tailor towards specific experimental needs^31^. For instance, pharmacological interventions with vasodilators are common practice in clinical NMP and have been demonstrated to be crucial for achieving microcirculatory homeostasis in other organs (i.e. livers)^32^. In heart perfusion, the administration of adenosine, a well-known vasodilator, in combination with low perfusion pressures seemed to reduce vascular resistance and completely avoid the formation of cardiac edema^33^. Hearts perfused with only Adenosine demonstrated statistically lower vascular resistance, when compared with non-treated hearts (Fig. 5A, *p* < 0.0001). Interestingly, the addition of Epinephrine into the Adenosine drip (Epi/Adenosine) caused a slight increase in vascular resistance, being statistically higher than Adenosine-only hearts (*p* = 0.0014) but still lower than no-treatment hearts (*p* = 0.0021). These differences in vascular resistance, however, were not associated with endothelial cell damage as the endothelial cell lining in the coronary arteries were retained irrespective of perfusion drip as seen in H&E images (Fig. 5G, J, M). Similarly, no myocyte bundle separation was observed in either ventricle of hearts perfused at low pressures with adenosine drip (Fig. 5H & I). Alternatively, despite causing less edema than high perfusion pressures, perfusion at low pressures, without Adenosine, were not enough to completely avoid edema formation as significant myocyte separation was present in the right ventricle (Fig. 5E) and slight separation in the left ventricle (Fig. 5F) of hearts perfused at low perfusion pressures without adenosine. The difference in the magnitude of bundle separation between the two ventricles may likely be attributed to several distinct characteristics that differentiate them. For instance, the increased thickness of the left ventricular wall results in higher vascular resistance, which helps prevent excessive flow into the left ventricle^34^. Additionally, studies have shown that left ventricular coronary perfusion is more effectively regulated through pressure-flow autoregulation than right ventricular perfusion, which may help reduce flow-induced damage^35^. Interestingly, inotrope (epinephrine) administration caused slight bundle separation in the left ventricle (Fig. 5L), despite the implementation of adenosine and low perfusion pressure, while no separation was present in the right ventricle (Fig. 5K). This bundle separation, or lack thereof, translated into hearts perfused with adenosine drip to gain the least percent weight, although non-statically significant, than hearts in the other two groups which demonstrated very similar changes in weight (Fig. 5B). Intriguingly, the use of Adenosine with (*p* = 0.0009) or without (*p* = 0.0087) the addition of Epinephrine caused grafts to experience a statistically higher oxygen uptake rate compared to acellular grafts perfused without treatment (Fig. 5D) while the adenylated energy charge remained statistically the same among all low-pressure hearts, regardless of pharmacological intervention (Fig. 5C). Additionally, the implementation of adenosine as a continuous drip resulted in the lowest secretion of inflammatory markers as, both ICAM (Fig. 7E) and IL-6 (Fig. 7F) where under the limit of detection and CXCL1 (Fig. 7G) was lower, although not statistically significant, than that detected in no Drip and Epinephrine.

**Fig. 6:**
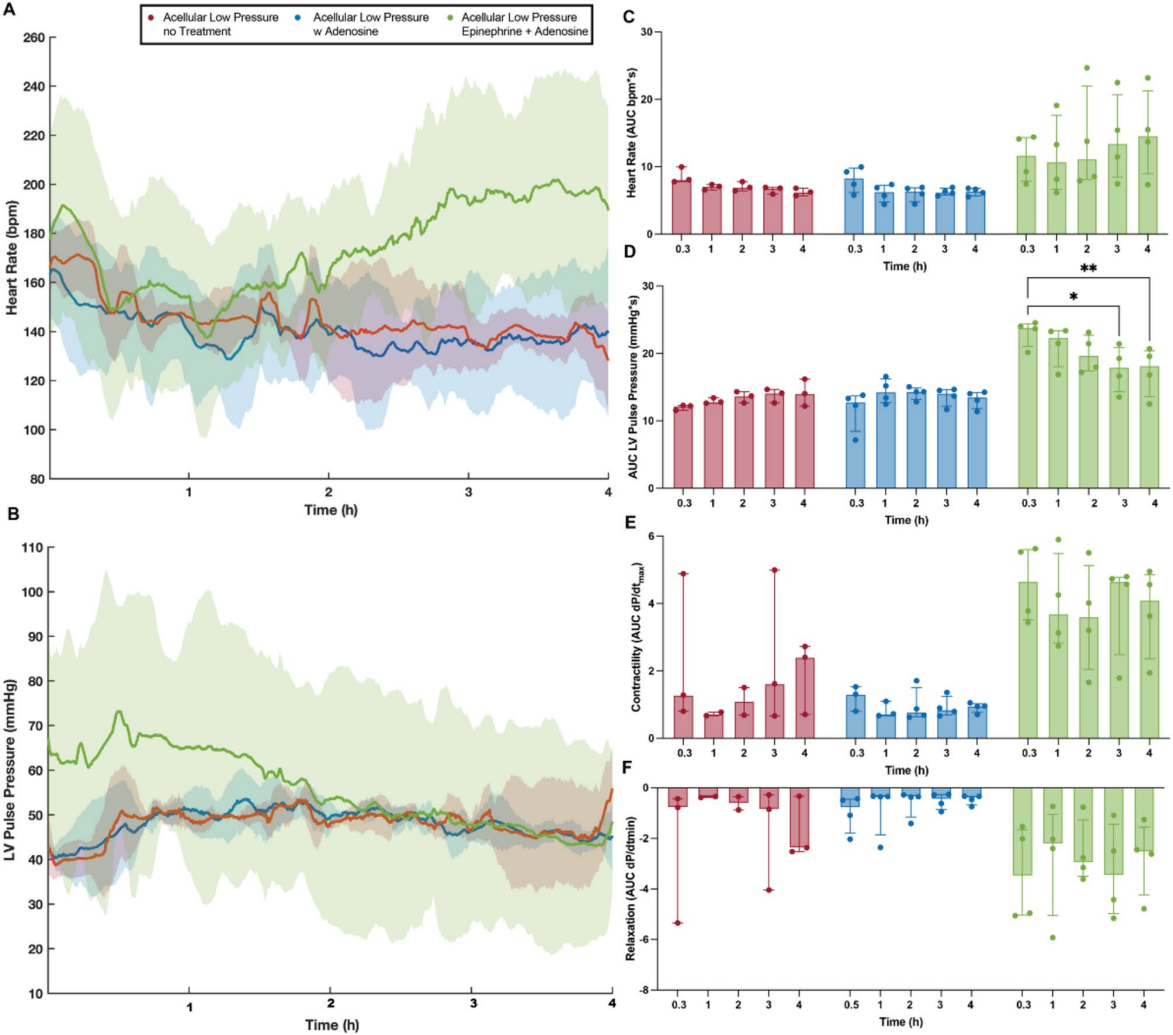
Influence of pharmacological treatment in cardiac functional markers. Heart rate, obtained from intraventricular balloon recordings. Solid line indicates median of the experimental replicates, the shaded area indicates interquartile range (**A**), maximum systolic pressure over time, denoted as left ventricular pulse pressure (LVPP). Solid line indicates median of the experimental replicates, the shaded area indicates interquartile range (**B**), area under the curve (AUC) of heart rate data for every hour of perfusion (**C**), the area under the LVPP curve (AUC) for every hour of perfusion (**D**), cardiac muscle contractility quantified from the maximum derivative of the pressure pulse (**E**), cardiac muscle relaxation quantified from the minimum derivative of the pressure pulse (**F**). Repeated measures two-way ANOVA and Tukey-Kramer HSD post-hoc analysis on standard least squares means. All data are expressed as median ± interquartile range.   * = p < 0.05, ** = p ≤ 0.01

### Continuous use of epinephrine during Langendorff perfusion leads to overtime loss of function and organ damage

**Fig. 7:**
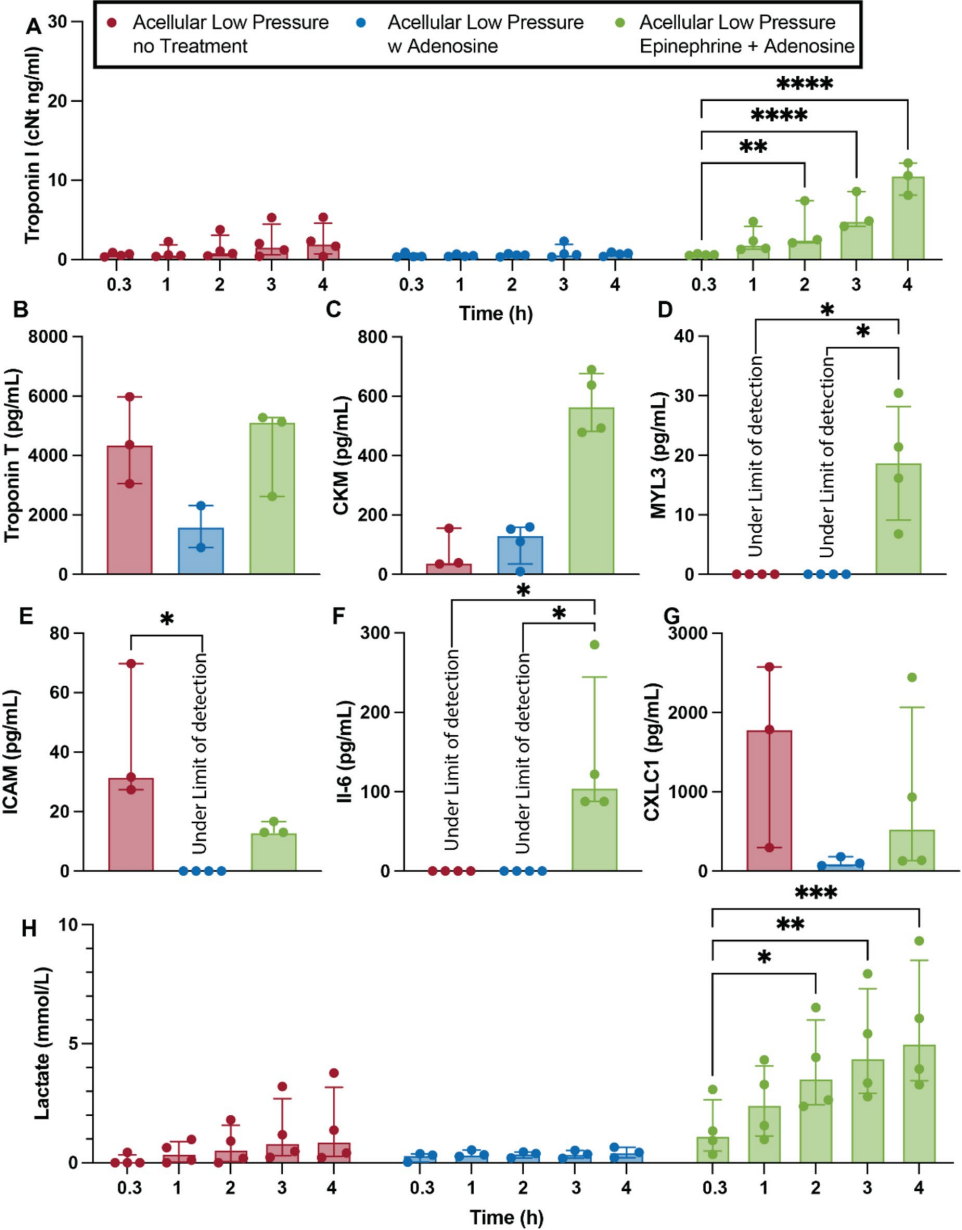
Influence of pharmacological treatment in cardiac injury and inflammatory markers. Concentration of Troponin I in the perfusate over time (**A**). Concentration of cardiac myocyte-associated proteins, a proxy for cardiac injury, quantified from the perfusate after 4 h (240 min) of perfusion, including Troponin T (**B**), creatine kinase – CKM (**C**), and myosin light chain 3 - MYL3 (**D**). Concentration of pro-inflammatory proteins including ICAM (**E**), IL-6 (**F**), and CXCL1 (**G**). Lactate accumulation over perfusion time (**H**). Repeated measures two-way ANOVA and Tukey-Kramer HSD post-hoc analysis on standard least squares means. All data are expressed as median ± interquartile range. * = *p* < 0.05, ** = *p* ≤ 0.01, *** = *p* ≤ 0.001, **** = *p* ≤ 0.0001.

Pharmacological interventions with inotropes are also common practice, both in experimental settings and clinical practice^36^. Constant infusion of epinephrine via a direct drip leads to statistical higher heart rate (Fig. 6A & C, *p* = 0.001), left ventricular pulse pressure (Fig. 6B & D, *p* < 0.0001), contractility (Fig. 6E, *p* = 0.0003) and relaxation (Fig. 6F, *p* = 0.018) than non-treated hearts when exposed to same perfusion pressures (30–35 mmHg). However, similar to hearts perfused at higher pressure, the extra cardiac demand induced by inotrope exposure resulted in overtime loss of cardiac function, with left ventricular pulse pressure being statistically lower at the beginning of the 3rd hour of perfusion (*p* = 0.0129) and thereafter (p_4h_ = 0.0074) than that recorded at the begging of perfusion (20 min). No loss in function was seen in no-treatment hearts (20 min vs. 4 h, *p* = 0.775) or hearts infused with adenosine (20 min vs. 4 h, *p* = 0.867). Commensurate with increased demand for LV pressure in this non-physiological setting, the use of inotropes resulted in increased release of cardiac myocyte-associated proteins with the accumulation of Troponin I in the perfusate being statistically higher than initial values as early as 2 h (Fig. 7A, *p* = 0.0031) into the perfusion time and thereafter (p_3h_ < 0.0001 and p_4h_ < 0.0001). Similarly, the concentration in the perfusate of CMK (Fig. 7C) and MYL3 (Fig. 7D, p_no-treatment_ = 0.064, p_Adenosine_ = 0.076) of hearts infused with inotropes were much higher than those found in no-treatment and Adenosine infused hearts. The concentration of Troponin T was slightly lower in Adenosine infused hearts, but not significantly (Fig. 7B). Similarly, a significant increase in lactate accumulation was seen in inotrope treated hearts with statistically higher levels being recorded as early as 2 h (*p* = 0.0128) into perfusion time (Fig. 7H, p_3h_ = 0.0011, p_4h_ = 0.0001), with no increase in lactate recorded in hearts perfused with or without adenosine-only.

## Discussion

Ex vivo heart perfusion has significantly advanced cardiovascular research, expanding the organ donor pool by enabling the transplantation of previously marginal hearts^37^. However, the impact of perfusion parameters on heart viability remains unclear. For instance, the perceived inefficiency of acellular perfusates in providing adequate oxygenation has fueled debate over the necessity of oxygen carriers during ex vivo perfusions^22,38^. The results in this manuscript challenge the notion of insufficient oxygen delivery by acellular perfusates, at least in the rodent model. Despite decreased oxygen consumption, likely caused by complete dependence on oxygen diffusion for oxygenation, no difference in adenylated energy charge (Fig. 1D) was observed between hearts perfused with or without oxygen carriers. This was the case even though the amounts of produced LV pressures varied widely among the hearts in the different perfusion groups (Fig. 2). This, combined with the observation that hearts perfused with acellular perfusate (i.e. less efficient oxygen delivery) at high pressures seem to produce slightly less LV pressures than those perfused cellularly (i.e. more efficient oxygen delivery) at similar pressures, suggests the possibility of a self-regulatory mechanism in which hearts produce the amount of LV pressure that allows the maintenance of energetic homeostasis. In effect, despite the lower efficiency of acellular perfusates in oxygen delivery, these results suggest the possibility that energetic demands are correspondingly decreased when perfused in retrograde mode regardless of the presence or absence of oxygen carriers.

The ability of acellular perfusates to provide sufficient oxygenation in the rodent model likely comes from the extremely high partial pressures of oxygen reached in the perfusate by artificial oxygenation – 450 ± 53 mmHg in acellular perfusions in this study – being over 4-fold higher than physiological oxygen partial pressures (75–100 mmHg)^39^. Furthermore, the oxygen saturation levels of these perfusates are not significantly increased by the addition of red blood cells, resulting in the partial pressure of oxygen of cellular perfusates (482 ± 73 mmHg) being very similar to that of acellular ones. It is important to highlight that while the high partial pressure of oxygen may ensure adequate oxygen delivery, it also raises concerns about potential risks associated with hyperoxia. Despite not being well characterized in hearts, some identified effects can negatively influence results and more importantly skew scientific conclusions if not taken into consideration. Hyperoxia has been reported to result in changes in heart rate, stroke volume, and cardiac output because of vasoconstriction, as well as heighten inflammatory response and cytotoxicity even after short periods of hyperoxia exposure^40–44^.

The addition of red blood cells, in combination with high perfusion pressures, seems ideal for experimental setups were maximizing cardiac demand is relevant. However, the use of these oxygen carriers in this particular setup appears to lead to a range of unfavorable effects. Red blood cells are major producers of lactate and are likely responsible for the progressive increase observed in hearts perfused with pRBCs (Fig. 1B)^45^. Experimentally, this increase in lactate can cause drops in perfusate pH due to lactic acidosis, though this can be easily countered with clinically used basic solutions^46^. A more severe consequence of this large increase in recorded lactate is its impact on the ability to assess heart health and viability. Despite growing evidence questioning its predictive power^47,48^, in-perfusate lactate levels are broadly used, both in-clinic and in experimental settings, as one of the few quantitative markers of organ health^49–56^. The inability to distinguish between lactate secreted by the heart due to poor perfusion or damage and that released by red blood cells undermines the decision-making power and may lead to inaccurate results and unfounded medical decisions.

In addition to lactate related issues, increased vascular resistance is another effect of using red blood cells in this experimental setup (Fig. 1C). The addition of red blood cells increases perfusate viscosity, which, in turn, raises the resistance within the vascular system^57^. Although this increase in resistance means target perfusion pressures are reached at lower flow rates, no evidence of underperfusion or ischemia was observed in hearts perfused with pRBCs. However, due to the non-physiological nature of perfusion setups, red blood cell lysis is unavoidable, leading to the release of red blood cell damage-associated molecular patterns (DAMPs) and red blood cell aggregation. Red blood cell DAMPs are endogenous molecules that are exclusively released upon red blood cell damage and are known to modulate significant immune system activation and may be responsible for the heightened inflammatory response observed in hearts perfused with pRBCs (Fig. 4E–G)^58–61^. Concerningly, this heightened inflammatory response is present when using isolated red blood cells alone, suggesting the inflammatory activation when using whole blood may be more severe, as both leukocytes and platelets are known orchestrators of immune response activation^62–64^.

A third plausible cause of increased inflammation in hearts perfused with pRBCs—and a probable reason why hearts perfused with pRBCs at low pressure show higher inflammatory response and higher indices of damage (Fig. 4A–D) than hearts in all other groups—is red blood cell aggregation. Red blood cell aggregation has been widely correlated to inflammation, unfavorable effects to tissue perfusion and suppression of nitric oxide synthetizing mechanisms^60,65,66^. It occurs when red blood cells are suspended in aqueous solutions containing large plasma proteins or polymers and exposed to low shear rates caused by low flows^67,68^. Both of these conditions are unfortunately well met in these experiments. The perfusate solution (Krebs-Henseleit) in these experiments was supplemented with bovine serum albumin (BSA) to provide an oncotic agent with the purpose of reducing heart edema. Although effective at reducing edema (Fig. 3I), BSA is a large plasma protein that likely facilitates aggregate formation. In addition, the lower-than-physiological flows (13 ± 2 mL/min in rodent) required to achieve even high perfusion pressures (8.3 ± 3.3 mL/min) likely contribute further towards red blood cell aggregation^69^. This aggregation is probably exacerbated by the even lower flows (4 ± 0.624 mL/min) in low pressure perfusions.

It is important to highlight that some of the negative effects of pRBCs perfusion observed in this study might be exacerbated by the comparatively small size of rodent vasculature, and some of these effects might either not be present or be less severe in larger mammalian hearts. Moreover, red blood cells or other types of oxygen carriers would likely be necessary to achieve working-mode perfusion in larger mammals. Although there are few studies achieving working-mode perfusion of rodent hearts with acellular perfusates this is likely achievable due to the absolute lower volume of myocardium in rodent hearts. The increased myocardial volume plus higher metabolic demand in large mammalian hearts may necessitate the use of oxygen carriers despite the potential negative effects^38,70–72^.

Additional to the effects caused by the lack/presence of red blood cells, the use of both pharmacological interventions (Epinephrine and/or Adenosine) utilized in this study also seemed to have considerable outcomes. Adenosine, a well-known vasodilator, is utilized in clinical ex vivo machine perfusion to aid in the stabilization and control of the aortic root pressure. It achieves this by dilating the coronary arteries and reducing coronary vascular resistance, ensuring proper blood flow during preservation^37,73^. Furthermore, recent clinical studies have shown a correlation between higher dosages of adenosine, likely needed to counteract increased coronary vascular resistance, and higher demand for ECMO implementation post-transplant^44^. In effect, the implementation of adenosine results in lower vascular resistance in all hearts treated with the agent (Figs. 1C and 5A), but also in a notably reduction of inflammatory responses (Figs. 4E-G and 7E–G). The anti-inflammatory role of adenosine in the systemic circulation has been widely reported, with notably effects in the inhibition of recruitment and activation of immune cells such as neutrophil and monocytes, as well as the reduction of thrombin-mediated proinflammatory responses in endothelial cells^74–76^. In the absence of the key players in inflammatory effects, the anti-inflammatory effects of adenosine may be attributed to the adenosine receptors expressed on endothelial cells, which upon exposure to ischemia and inflammation inhibit the release and expression of cytokines and adhesion molecules^76,77^. These results suggest that adenosine not only optimizes hemodynamic parameters but may also contribute to a more stable and less inflammatory perfusion environment, further enhancing its utility in cardiac perfusion protocols.

The use of epinephrine, on the other hand, appears to be less beneficial in the ex vivo perfusion setting. Epinephrine is reportedly used in ex vivo machine perfusion to help regulate aortic pressures and coronary flow, as it increases diastolic time, heart rate and to maintain physiological levels of circulating catecholamines^78–80^. However, the long-term use of epinephrine has been associated with myocardial toxicity, apoptosis and damage to the arterial walls both in vivo and ex vivo^79,81^. Although 4 h is not typically considered long-term in the realm of physiology, some negative effects were observed in hearts exposed to a continuous drip of epinephrine. For instance, increased edema was observed in the left ventricle of these hearts (Fig. 5L), an effect either not present or in a lesser degree in other hearts perfused at low pressure, possibly caused by deterioration of the arterial walls. Similarly, a gradual loss of function was noted (Fig. 6), although likely caused by the increased cardiac demand in a non-physiological setting rather than apoptosis, as there is no evidence of myocyte death in the histology images (Fig. 5E–M). Lastly, is important to point out that although not evident in the results of this study exposure to non-physiological low perfusion pressures could potentially result in pathophysiological response within the organ and would benefit from further research.

### Limitations

Cardiac ex vivo machine perfusion involves a wide range of protocol variables, each of which can significantly influence outcomes (e.g., perfusion pressure, flow rate, temperature, oxygenation levels, perfusate composition, pharmacological interventions, etc.). Since the aim of this study was to identify the optimal combination of these variables for a limited number of experimental scenarios based on specific scientific needs (i.e., assessment vs. preservation), multiple variables were altered between experimental groups. This complicates the interpretation of the effects of individual variables and makes it difficult to isolate the contribution of any single factor. Regardless, the results in this study provide an important knowledge base regarding the effects that the combination of perfusion variables have on rodent cardiac graft outcomes during Langendorff. It should also be noted that there are important anatomical, physiological, and metabolic differences between rodent and human hearts, thereby limiting the direct applicability of these findings to human cardiac grafts during perfusion. Nonetheless, this work highlights the importance of perfusion protocol tailoring and can provide a starting point for perfusion research employing more complex models, such as larger mammals commonly employed in experimental transplantation, including pig or sheep. These larger animal models may help ameliorate the translational gap between rodent and human research and lead to more applicable protocol development for human heart perfusion and assessment.

## Conclusion

This study highlights the impact of different perfusion parameters and pharmacological interventions on rodent organ viability, function, and injury in ex vivo machine perfusion. It emphasizes the need to tailor these parameters to suit experimental goals. For longer perfusion times, lower pressures are ideal to minimize damage (e.g., gene editing, organ preservation, drug treatments), while higher pressures and oxygen carriers are better for cardiac assessments. Immunogenic effects can be adjusted with red blood cells or whole blood for higher responses, or acellular perfusates and adenosine for lower ones. Similar to these examples, the myriads of perfusion parameters during Langendorff can be modified and selected to create experimental conditions that would enhance the scientific results. As a result, this work contributes to the broader cardiovascular research community by providing valuable insights into the complex factors influencing heart ex vivo machine perfusion. It offers a foundation for refining protocols that can be applied to both rodent and eventually larger mammalian models, while highlighting the importance of thoughtful selection of perfusion parameters.

## Data Availability

All data generated or analyzed during this study are included in this published article.

## References

[1] Organization, W. H. Cardiovascular diseases (CVDs) (2021). https://www.who.int/news-room/fact-sheets/detail/cardiovascular-diseases-(cvds).

[2] Dharmavaram, N. et al. National trends in heart donor usage rates: are we efficiently transplanting more hearts? *J. Am. Heart Assoc.* **10** (15), e019655 (2021). 34315285 10.1161/JAHA.120.019655 PMC8475695

[3] Tsao, C. W. et al. Heart disease and stroke Statistics-2023 update: a report from the American heart association. *Circulation* **147** (8), e93–e621 (2023). 36695182 10.1161/CIR.0000000000001123 PMC12135016

[4] Lopez, A. D. & Adair, T. Is the long-term decline in cardiovascular-disease mortality in high-income countries over? Evidence from National vital statistics. *Int. J. Epidemiol.* **48** (6), 1815–1823 (2019). 31378814 10.1093/ije/dyz143

[5] Rosenzweig, A. The growing importance of basic models of cardiovascular disease. *Circ. Res.* **130** (12), 1743–1746 (2022). 35679368 10.1161/CIRCRESAHA.122.321368 PMC9202074

[6] Onodi, Z. et al. Systematic transcriptomic and phenotypic characterization of human and murine cardiac myocyte cell lines and primary cardiomyocytes reveals serious limitations and low resemblances to adult cardiac phenotype. *J. Mol. Cell. Cardiol.* **165**, 19–30 (2022). 34959166 10.1016/j.yjmcc.2021.12.007

[7] Hearse, D. J. & Sutherland, F. J. Experimental models for the study of cardiovascular function and disease. *Pharmacol. Res.* **41** (6), 597–603 (2000). 10816328 10.1006/phrs.1999.0651

[8] Savoji, H. et al. Cardiovascular disease models: a game changing paradigm in drug discovery and screening. *Biomaterials* **198**, 3–26 (2019). 30343824 10.1016/j.biomaterials.2018.09.036 PMC6397087

[9] Bell, R. M., Mocanu, M. M. & Yellon, D. M. Retrograde heart perfusion: the Langendorff technique of isolated heart perfusion. *J. Mol. Cell. Cardiol.* **50** (6), 940–950 (2011). 21385587 10.1016/j.yjmcc.2011.02.018

[10] Mackin, C. et al. Intravenous Amiodarone and Sotalol impair contractility and cardiac output, but procainamide does not: A Langendorff study. *J. Cardiovasc. Pharmacol. Ther.* **24** (3), 288–297 (2019). 30497293 10.1177/1074248418810811 PMC6980379

[11] Li, Q. et al. Multiple mass isotopomer tracing of acetyl-CoA metabolism in Langendorff-perfused rat hearts: channeling of acetyl-CoA from pyruvate dehydrogenase to carnitine acetyltransferase. *J. Biol. Chem.* **290** (13), 8121–8132 (2015). 25645937 10.1074/jbc.M114.631549 PMC4375469

[12] Jungen, C. et al. Impact of intracardiac neurons on cardiac electrophysiology and arrhythmogenesis in an ex vivo Langendorff system. *J. Vis. Exp.* **2018**, 135 (2018). 10.3791/57617 PMC6101334 29889210

[13] Zimmer, H. G. The isolated perfused heart and its pioneers. *News Physiol. Sci.* **13**, 203–210 (1998). 11390791 10.1152/physiologyonline.1998.13.4.203

[14] Pendexter, C. A. et al. Modified Langendorff perfusion for extended perfusion times of rodent cardiac grafts. *JoVE* **208**, e66815 (2024). 10.3791/66815 PMC11771952 38949317

[15] Testai, L. et al. Cardioprotective effects of different flavonoids against myocardial ischaemia/reperfusion injury in Langendorff-perfused rat hearts. *J. Pharm. Pharmacol.* **65** (5), 750–756 (2013). 23600393 10.1111/jphp.12032

[16] Matsuura, H. et al. Positive inotropic effects of ATP released via the Maxi-Anion channel in Langendorff-Perfused mouse hearts subjected to Ischemia-Reperfusion. *Front. Cell. Dev. Biol.* **9**, 597997 (2021). 33553176 10.3389/fcell.2021.597997 PMC7859278

[17] Louradour, J. et al. Simultaneous assessment of mechanical and electrical function in Langendorff-perfused ex-vivo mouse hearts. *Front. Cardiovasc. Med.* **10**, 1293032 (2023). 38028448 10.3389/fcvm.2023.1293032 PMC10663365

[18] King, D. R. et al. Reevaluating methods reporting practices to improve reproducibility: an analysis of methodological rigor for the Langendorff whole heart technique. *Am. J. Physiol. Heart Circ. Physiol.* **323** (3), H363–H377 (2022). 35749719 10.1152/ajpheart.00164.2022 PMC9359653

[19] de Vries, R. J. et al. Supercooling extends preservation time of human livers. *Nat. Biotechnol.* **37** (10), 1131–1136 (2019). 31501557 10.1038/s41587-019-0223-y PMC6776681

[20] Tessier, S. N. et al. Partial freezing of rat livers extends preservation time by 5-fold. *Nat. Commun.* **13** (1), 4008 (2022). 35840553 10.1038/s41467-022-31490-2 PMC9287450

[21] Bruinsma, B. G. et al. Metabolic profiling during ex vivo machine perfusion of the human liver. *Sci. Rep.* **6**, 22415 (2016). 26935866 10.1038/srep22415 PMC4776101

[22] Noly, P. E. et al. Assessment of ex vivo murine biventricular function in a Langendorff model. *J. Vis. Exp.* **2022**, 190 (2022). 10.3791/64384 PMC10226723 36622020

[23] White, C. W. et al. A whole blood-based perfusate provides superior preservation of myocardial function during ex vivo heart perfusion. *J. Heart Lung Transpl.* **34** (1), 113–121 (2015). 10.1016/j.healun.2014.09.021 25447577

[24] Abel, R. M. & Reis, R. L. Effects of coronary blood flow and perfusion pressure on left ventricular contractility in dogs. *Circ. Res.* **27** (6), 961–971 (1970). 4992167 10.1161/01.res.27.6.961

[25] Arnold, G., Morgenstern, C. & Lochner, W. The autoregulation of the heart work by the coronary perfusion pressure. *Pflugers Arch.* **321** (1), 34–55 (1970). 5529741 10.1007/BF00594121

[26] Iwamoto, T., Bai, X. J. & Downey, H. F. Coronary perfusion related changes in myocardial contractile force and systolic ventricular stiffness. *Cardiovasc. Res.* **28** (9), 1331–1336 (1994). 7954641 10.1093/cvr/28.9.1331

[27] Opie, L. H. Coronary flow rate and perfusion pressure as determinants of mechanical function and oxidative metabolism of isolated perfused rat heart. *J. Physiol.* **180** (3), 529–541 (1965). 5846791 10.1113/jphysiol.1965.sp007715 PMC1357401

[28] Bui, T. M., Wiesolek, H. L. & Sumagin, R. ICAM-1: A master regulator of cellular responses in inflammation, injury resolution, and tumorigenesis. *J. Leukoc. Biol.* **108** (3), 787–799 (2020). 32182390 10.1002/JLB.2MR0220-549R PMC7977775

[29] Tanaka, T., Narazaki, M. & Kishimoto, T. IL-6 in inflammation, immunity, and disease. *Cold Spring Harb Perspect. Biol.* **6** (10), a016295 (2014). 25190079 10.1101/cshperspect.a016295 PMC4176007

[30] Sawant, K. V. et al. Chemokine CXCL1 mediated neutrophil recruitment: role of glycosaminoglycan interactions. *Sci. Rep.* **6**, 33123 (2016). 27625115 10.1038/srep33123 PMC5021969

[31] Buchwald, J. E. et al. Therapeutics administered during ex vivo liver machine perfusion: an overview. *World J. Transpl.* **10** (1), 1–14 (2020). 10.5500/wjt.v10.i1.1 PMC7031625 32110510

[32] Nassar, A. et al. Role of vasodilation during normothermic machine perfusion of DCD Porcine livers. *Int. J. Artif. Organs*. **37** (2), 165–172 (2014). 24619899 10.5301/ijao.5000297

[33] Guieu, R. et al. Adenosine and the cardiovascular system: the good and the bad. *J. Clin. Med.* **9**, 5 (2020). 10.3390/jcm9051366 PMC7290927 32384746

[34] Carabello, B. A. Understanding coronary blood flow: the wave of the future. *Circulation* **113** (14), 1721–1722 (2006). 16606800 10.1161/CIRCULATIONAHA.105.617183

[35] Crystal, G. J. & Pagel, P. S. Right ventricular perfusion: physiology and clinical implications. *Anesthesiology* **128** (1), 202–218 (2018). 28984631 10.1097/ALN.0000000000001891

[36] Pinnelas, R. & Kobashigawa, J. A. Ex vivo normothermic perfusion in heart transplantation: a review of the TransMedics((R)) organ care system. *Future Cardiol.* **18** (1), 5–15 (2022). 34503344 10.2217/fca-2021-0030

[37] Bryner, B. S., Schroder, J. N. & Milano, C. A. Heart transplant advances: ex vivo organ-preservation systems. *JTCVS Open.* **8**, 123–127 (2021). 36004090 10.1016/j.xjon.2021.04.020 PMC9390583

[38] DeWitt, E. S., Black, K. J. & Kheir, J. N. Rodent working heart model for the study of myocardial performance and oxygen consumption. *J. Vis. Exp.* **2016**, 114 (2016). 10.3791/54149 PMC5091847 27584550

[39] Malatesha, G. et al. Comparison of arterial and venous pH, bicarbonate, PCO2 and PO2 in initial emergency department assessment. *Emerg. Med. J.* **24** (8), 569–571 (2007). 17652681 10.1136/emj.2007.046979 PMC2660085

[40] Rodgers, J. L. et al. Impact of hyperoxia on cardiac pathophysiology. *J. Cell. Physiol.* **234** (8), 12595–12603 (2019). 30652312 10.1002/jcp.28136 PMC13484087

[41] Sinski, M. et al. Deactivation of carotid body chemoreceptors by hyperoxia decreases blood pressure in hypertensive patients. *Hypertens. Res.* **37** (9), 858–862 (2014). 24804611 10.1038/hr.2014.91

[42] Smit, B. et al. Hemodynamic effects of acute hyperoxia: systematic review and meta-analysis. *Crit. Care*. **22** (1), 45 (2018). 29477145 10.1186/s13054-018-1968-2 PMC6389225

[43] Hafner, C. et al. Hyperoxia induces inflammation and cytotoxicity in human adult cardiac myocytes. *Shock* **47** (4), 436–444 (2017). 27648689 10.1097/SHK.0000000000000740

[44] Amesz, J. H. et al. Myocardial oxygen handling and metabolic function of ex-situ perfused human hearts from circulatory death donors. *JHLT Open.* **6**, 100159 (2024). 40145048 10.1016/j.jhlto.2024.100159 PMC11935508

[45] Lee, T. Y. Lactate: a multifunctional signaling molecule. *Yeungnam Univ. J. Med.* **38** (3), 183–193 (2021). 33596629 10.12701/yujm.2020.00892 PMC8225492

[46] Seheult, J., Fitzpatrick, G. & Boran, G. Lactic acidosis: an update. *Clin. Chem. Lab. Med.* **55** (3), 322–333 (2017). 27522622 10.1515/cclm-2016-0438

[47] Cernic, S. et al. Lactate during ex-situ heart perfusion does not predict the requirement for mechanical circulatory support following donation after circulatory death (DCD) heart transplants. *J. Heart Lung Transpl.* **41** (9), 1294–1302 (2022). 10.1016/j.healun.2022.02.003 35811221

[48] Saemann, L. et al. Monitoring of perfusion quality and prediction of donor heart function during ex-vivo machine perfusion by myocardial microcirculation versus surrogate parameters. *J. Heart Lung Transpl.* **40** (5), 387–391 (2021). 10.1016/j.healun.2021.02.013 33726982

[49] Hamed, A. et al. 19: serum lactate is a highly sensitive and specific predictor of post cardiac transplant outcomes using the organ care system. *J. Heart Lung Transplantation*. **28** (2), S71 (2009).

[50] Ardehali, A. et al. Ex-vivo perfusion of donor hearts for human heart transplantation (PROCEED II): a prospective, open-label, multicentre, randomised non-inferiority trial. *Lancet* **385** (9987), 2577–2584 (2015). 25888086 10.1016/S0140-6736(15)60261-6

[51] Dhital, K. K. et al. Adult heart transplantation with distant procurement and ex-vivo preservation of donor hearts after circulatory death: a case series. *Lancet* **385** (9987), 2585–2591 (2015). 25888085 10.1016/S0140-6736(15)60038-1

[52] Dhital, K. K., Chew, H. C. & Macdonald, P. S. Donation after circulatory death heart transplantation. *Curr. Opin. Organ. Transpl.* **22** (3), 189–197 (2017). 10.1097/MOT.0000000000000419 28379853

[53] Qin, G. et al. Machine perfusion for human heart preservation: a systematic review. *Transpl. Int.* **35**, 10258 (2022). 35401041 10.3389/ti.2022.10258 PMC8983812

[54] van Suylen, V. et al. Oxygenated machine perfusion at room temperature as an alternative for static cold storage in Porcine donor hearts. *Artif. Organs*. **46** (2), 246–258 (2022). 34633676 10.1111/aor.14085 PMC9298357

[55] Saemann, L. et al. Reconditioning of circulatory death hearts by ex-vivo machine perfusion with a novel HTK-N preservation solution. *J. Heart Lung Transpl.* **40** (10), 1135–1144 (2021). 10.1016/j.healun.2021.07.009 34420849

[56] Barajas, M. B. & Levy, R. J. Modified technique for the use of neonatal murine hearts in the Langendorff Preparation. *J. Vis. Exp.* **2022**, 181 (2022). 10.3791/63349 PMC12909778 35311818

[57] Trejo-Soto, C. & Hernandez-Machado, A. Normalization of blood viscosity according to the hematocrit and the shear rate. *Micromachines (Basel)*, **13**, 3 (2022). 10.3390/mi13030357 PMC8954080 35334649

[58] Zhou, H. L., Jiang, X. Z. & Ventikos, Y. Role of blood flow in endothelial functionality: a review. *Front. Cell. Dev. Biol.* **11**, 1259280 (2023). 37905167 10.3389/fcell.2023.1259280 PMC10613523

[59] Jeney, V. Pro-Inflammatory actions of red blood Cell-Derived damps. *Exp. Suppl.* **108**, 211–233 (2018). 30536173 10.1007/978-3-319-89390-7_9

[60] Ami, R. B. et al. Parameters of red blood cell aggregation as correlates of the inflammatory state. *Am. J. Physiol. Heart Circ. Physiol.* **280** (5), pH1982–pH1988 (2001). 10.1152/ajpheart.2001.280.5.H1982 11299197

[61] Kato, J. & Svensson, C. I. Role of extracellular damage-associated molecular pattern molecules (DAMPs) as mediators of persistent pain. *Prog Mol. Biol. Transl Sci.* **131**, 251–279 (2015). 25744676 10.1016/bs.pmbts.2014.11.014

[62] Chen, L. et al. Inflammatory responses and inflammation-associated diseases in organs. *Oncotarget* **9** (6), 7204–7218 (2018). 29467962 10.18632/oncotarget.23208 PMC5805548

[63] Bo, Y. et al. The role of platelets in central hubs of inflammation: a literature review. *Med. (Baltim).* **103** (19), e38115 (2024). 10.1097/MD.0000000000038115 PMC11081549 38728509

[64] Hatami, S. et al. Immunity and stress responses are induced during ex situ heart perfusion. *Circ. Heart Fail.* **13** (6), e006552 (2020). 32498623 10.1161/CIRCHEARTFAILURE.119.006552

[65] Lazari, D. et al. The relationship between aggregation and deformability of red blood cells in health and disease. *Front. Physiol.* **11**, 288 (2020). 32351399 10.3389/fphys.2020.00288 PMC7174766

[66] Baskurt, O. K. et al. Modulation of endothelial nitric oxide synthase expression by red blood cell aggregation. *Am. J. Physiol. Heart Circ. Physiol.* **286** (1), H222–H229 (2004). 14512280 10.1152/ajpheart.00532.2003

[67] Baskurt, O. K. & Meiselman, H. J. Erythrocyte aggregation: basic aspects and clinical importance. *Clin. Hemorheol Microcirc*. **53** (1–2), 23–37 (2013). 22975932 10.3233/CH-2012-1573

[68] Reinhart, W. H., Piety, N. Z. & Shevkoplyas, S. S. Influence of red blood cell aggregation on perfusion of an artificial microvascular network. *Microcirculation* **24**, 5 (2017). 10.1111/micc.12317 PMC5357595 27647727

[69] Spear, K. L., Koerner, J. E. & Terjung, R. L. Coronary blood flow in physically trained rats. *Cardiovasc. Res.* **12** (3), 135–143 (1978). 647717 10.1093/cvr/12.3.135

[70] Aasum, E. et al. Age-dependent changes in metabolism, contractile function, and ischemic sensitivity in hearts from Db/db mice. *Diabetes* **52** (2), 434–441 (2003). 12540618 10.2337/diabetes.52.2.434

[71] Pedersen, T. M. et al. Isolated perfused working hearts provide valuable additional information during phenotypic assessment of the diabetic mouse heart. *PLoS One*. **13** (10), e0204843 (2018). 30273374 10.1371/journal.pone.0204843 PMC6166959

[72] Kearns, M. J. et al. A rodent model of cardiac donation after circulatory death and novel biomarkers of cardiac viability during ex vivo heart perfusion. *Transplantation* **101** (8), e231–e239 (2017). 28505025 10.1097/TP.0000000000001815

[73] Dang Van, S. et al. Ex vivo perfusion of the donor heart: preliminary experience in high-risk transplantations. *Arch. Cardiovasc. Dis.* **114** (11), 715–726 (2021). 34620574 10.1016/j.acvd.2021.07.003

[74] Hasko, G. & Cronstein, B. Regulation of inflammation by adenosine. *Front. Immunol.* **4**, 85 (2013). 23580000 10.3389/fimmu.2013.00085 PMC3619132

[75] Hassanian, S. M., Dinarvand, P. & Rezaie, A. R. Adenosine regulates the Proinflammatory signaling function of thrombin in endothelial cells. *J. Cell. Physiol.* **229** (9), 1292–1300 (2014). 24477600 10.1002/jcp.24568 PMC4037378

[76] Blackburn, M. R. et al. Adenosine receptors and inflammation. *Handb. Exp. Pharmacol.* **193**, 215–269 (2009). 10.1007/978-3-540-89615-9_8 19639284

[77] Bouma, M. G., van den Wildenberg, F. A. & Buurman, W. A. Adenosine inhibits cytokine release and expression of adhesion molecules by activated human endothelial cells. *Am. J. Physiol.* **270** (2 Pt 1), C522–C529 (1996). 8779915 10.1152/ajpcell.1996.270.2.C522

[78] Beuth, J. et al. New strategies to expand and optimize heart donor pool: ex vivo heart perfusion and donation after circulatory death: a review of current research and future trends. *Anesth. Analg*. **128** (3), 406–413 (2019). 30531220 10.1213/ANE.0000000000003919

[79] Overgaard, C. B. & Dzavik, V. Inotropes and vasopressors: review of physiology and clinical use in cardiovascular disease. *Circulation* **118** (10), 1047–1056 (2008). 18765387 10.1161/CIRCULATIONAHA.107.728840

[80] Messer, S., Ardehali, A. & Tsui, S. Normothermic donor heart perfusion: current clinical experience and the future. *Transpl. Int.* **28** (6), 634–642 (2015). 24853906 10.1111/tri.12361

[81] Singh, K. et al. Adrenergic regulation of cardiac myocyte apoptosis. *J. Cell. Physiol.* **189** (3), 257–265 (2001). 11748583 10.1002/jcp.10024

